# Supplementary material for: MetaRibo-Seq measures translation in microbiomes
Source: Nat Commun. 2020 Jun 29;11:3268. doi: 10.1038/s41467-020-17081-z (PMC7324362; doi:10.1038/s41467-020-17081-z)
Supplement: Supplementary file 10 — Supplementary Data 7 [file 41467_2020_17081_MOESM10_ESM.zip › File2/Confidence_High_Taxonomy/227218_out.krona.html]

Javascript must be enabled to view this page.

members
magnitude
magnitudeUnassigned
count
unassigned
taxon
rank

227218\_out

2


SRS143070\_contig\_number\_contig-100\_18004.62383
1

1
2
superkingdom

phylum
1239
1

class
186801
1

186802
1
order

1
186803
family

genus
1432051
1

species

SRS019068\_contig\_number\_40298
1
2292348
